# Lactic acid microflora of the gut of snail *Cornu aspersum*

**DOI:** 10.1080/13102818.2014.947071

**Published:** 2014-10-21

**Authors:** Zdravka Koleva, Ivaylo Dedov, Joana Kizheva, Roxana Lipovanska, Penka Moncheva, Petya Hristova

**Affiliations:** ^a^ Faculty of Biology, Sofia University ‘St. Kliment Ohridski’, Sofia, Bulgaria; ^b^ Institute of Biodiversity and Ecosystem Research, Bulgarian Academy of Sciences, Sofia, Bulgaria

**Keywords:** gut, snail, lactic acid bacteria, *Lactobacillus*, 16S-23S rDNA PCR, sequencing of 16S rDNA

## Abstract

The intestinal lactic acid microflora of the edible snail *Cornu aspersum* was studied by culture-based methods and was phenotypically and molecularly characterized. The antibacterial activity of lactic acid bacteria (LAB) isolates was investigated. Snails in different stages of development were collected from farms located in several regions of Bulgaria. One hundred twenty-two isolates, belonging to the group of LAB, were characterized morphologically and were divided into four groups. Representative isolates from each morphological type were subjected to phenotypic characterization and molecular identification. The snail gut lactic acid microflora was composed by *Enterococcus* (17 isolates), *Lactococcus* (12 isolates), *Leuconostoc* (7 isolates), *Lactobacillus* (18 isolates) and *Weissella* (1 isolate). The species affiliation of *Lactococcus lactis* (12), *Leuconostoc mesenteroides* (4) and *Lactobacillus plantarum* (2) was confirmed by species-specific primers. The *Lactobacillus* isolates were identified by sequence analysis of 16S rDNA as *Lactobacillus brevis* (12), *L. plantarum* (2), *Lactobacillus graminis* (1) and *Lactobacillus curvatus* (3). The species *L. brevis*, *L. graminis* and *L. curvatus* were found in snails in a phase of hibernation, whereas *L. plantarum* was identified both in active and hibernation phases. Antibacterial activity (bacteriocine-like) was shown only by one strain of *L. mesentereoides* P4/8 against *Propionibacterium acnes*. The present study showed that the LAB are a component of the microbial communities in the snail digestive system. This is the first report on *Lactobacillus* strains detected in the gut of *C. aspersum*.

## Introduction

Lactic acid bacteria (LAB) are an important part of the microbial population in the digestive tract of many animal species including pigs, fowls, rodents, chicken, horses, gastropods and insects, and play a significant role in maintaining the ecological equilibrium between the different species of microorganisms inhabiting these environments. This microbiota could also participate in the digestive process (fermentation) and the energy supply (L-lactate, acetate).[1] Terrestrial gastropods, as herbivore, eat fresh plants with high protein and calcium contents [2] and participate in the decomposition of leaf litter.[3] Their extraordinary efficiency in plant digestion (60%–80%) depends predominantly on the metabolitic activities of the intestinal microflora.[1] It was presumed that the alimentary tract is a major component of interaction between an animal's ecosystem and its physiology. The gut health of farm animals is closely related to the microbial balance of the intestinal flora. The diversity of bacterial microflora in the intestine of edible snails *Cornu aspersum* (Syn: *Helix aspersa*) and *Helix pomatia* has been studied by culture-based methods, 16S rRNA sequence analyses and phenotypic characterizations.[1] The bacterial species in the snail gut were arranged into two taxa: γ-Proteobacteria (*Buttiauxella*, *Citrobacter*, *Enterobacter*, *Kluyvera*, *Obesumbacterium*, *Raoultella*) and the Firmicutes (*Enterococcus*, *Lactococcus* and *Clostridium*). These genera are mostly assigned to enteric environments or to phyllosphere, data in favour of culturing snails in contact with soil and plants.[1] However, few studies investigated the presence and relative roles of LAB in food digestion and host nutrition. *Enterococcus casseliflavus* was already described as a dominant Gram-positive species in the gut of *C. aspersum* and was generally assigned to ‘enteric’ environments. This prevalence was explained by Charrier et al. [4] with the balance between the physico-chemical conditions in the snail intestine and the environmental requirements of epiphyte enterococci (growth at a pH > 5.0, a high humidity, temperature of 10 °C). Furthermore, the occurrence of active pullulanases in *E. casseliflavus* suggests culturing *C. aspersum* with amylopectin-rich cereals (maize, rice and sorghum) and to favour plant decaying – infestation by pullulan rich fungi, like basidiomycetes, part of gastropod food.[5] The presence of lactococci in the snail gut could be related with production of lactic acid. Moreover, *Lactococcus lactis* possesses a surface protein (HtrA), which is a key factor in the response to specific stress conditions [6] and significant for the survival of bacteria in the snail gut during hibernation. The presence of these two genera in the snail's intestine suggests that lactic acid might play an important role during the digestive process in the snail. Lactic acid has also been found to be a stimulatory response in a marine gastropod *Nassarius obsoletus*.[7] The occurrence of epiphyte enterococci as the dominant lactic bacterium in the snail's intestine, and not lactobacilli such as *Lactobacillus plantarum* also widespread on decaying plants is interesting.[8] Currently, there are no publications demonstrating the presence of other plant associated LAB (as *Lactobacillus* and *Leuconostoc*) in the intestinal tract of snails.

The aim of the present work was to isolate, cultivate, characterize and identify by phenotypic and genotypic methods the LAB associated with the snail's gut in order to improve the understanding of this microbial environment. *In vitro* determination of antimicrobial spectra against the bacterial and human foodborne pathogen was also the object of this study.

## Materials and methods

### Snail intestinal tract sampling

Six samples of *C. aspersum (Gastropoda, Pulmonata*) in different physiological stages were taken from various private snail farms in Bulgaria. All investigated samples were kept under starvation for approximately two weeks. The shells of snails were sterilized with ethanol (70 °C) and were removed aseptically. Snails were washed with sterile physiological solution and were dissected *in vivo* under aerobic conditions. The whole intestinal tract (esophagus to rectum) was aseptically handled to avoid contamination from external surface of the snail's body. The samples were used to obtain homogenates in physiological solution (0.85% w/w NaCl).

### Culture media

The aliquots (100 μL) of the different dilutions of the gut samples were spread in duplicate onto MRS and M17 agar medium (Merck). The plates were incubated at 28 and 37 °C for 48–72 hours in anaerobic conditions (Anaerocult, BioMerieux) in anoxic jars. The number of colony-forming units (CFU) per mL was counted after the incubation period.

### Phenotypic characterization of LAB

The morphology of the colonies forming after inoculation and cultivation on the media with sample dilutions was studied. Distinct colonies, which possessed different morphology, that were catalase and oxydase negative and consisted of Gram-positive cells were picked and further studied. Carbohydrate fermentation patterns (API CH50, API Strep 20, Biomerieux) and phenotypic identification according to basic biochemical tests were carried out for representative isolates.[9] All strains were stored as 40% glycerol stock cultures at −20 °C.

### Molecular differentiation of LAB

Molecular differentiation of isolated LAB at the genus level was carried out by polymerase chain reaction (PCR) amplification of 16S-23S ITS rDNA with the universal primers 16S/4 and 23S/7 as previously described.[10] Species grouping and species identification were performed by a two-step multiplex PCR according Song et al.[11] The genus and species affiliation of the strains obtained by the above methods was confirmed by PCR amplification with genus- and species-specific primer pairs – LbLMA1/R16-1,[10] Lpla2/Lpla-3,[11] LmeF/LmeR, and LlaF/LlaR,[12] respectively. The primers CA1/CA2, Efm1/Efm2 and Efs1/Efs2 were applied for differentiation and identification of *E. casseliflavus*,[13] *Enterococcus faecium* and *Enterococcus faecalis*,[14] respectively.

Total DNA of selected LAB strains was extracted from overnight culture on MRS medium with DNA kit prep GEM^TM^ Bacteria (ZyGEM). PCRs were performed in a total volume 25 μL containing 6.5 μL VWR Red *Taq* polymerase master Mix (VWr, Denmark), 1 μL of each primer (0.3 μmol/L concentration), 1 μL DNA (ca. 50 ng) and 15.5 μL H_2_O. PCRs were carried out in a thermocycler TC 312 (Techne). The reaction conditions were as follows: an initial denaturation step at 94 °C for 5 min, followed by 25 cycles at 94 °C for 45 s, 58 °C, 55 °C or 52 °C (according primers specificity) for 45 s, extension step at 72 °C for 45 s, and a final extension step for 7 min at 72 °C. The PCR products (aliquots of 5–10 μL) were resolved electrophoretically in 1.5% agarose gels (Agarose, DNA grade, Electran) in TBE 10× buffer at 100 V for 45 min. Gels were stained with ethidium bromide (5μg mL^−1^) and the bands were visualized under ultraviolet illumination at 254 nm. A 100 bp DNA Ladder was used as molecular mass marker (GeneRuler100 bp, 0.5μL/mL).

### 16S rRNA sequencing

The respective 16S rDNA genes were amplified by PCR using the universal primers 9F and 1542R.[15] The PCR products were purified and the DNA sequencing was carried out by Macrogen Services. Sequences were compared and aligned with those from the GenBank database using the BLAST program of the National Center for Biotechnology Information (NCBI; http://www. ncbi.nlm.nih.gov) network server.

### Antimicrobial activity of LAB

The antimicrobial activity of isolated LAB was determined by the agar overlay method.[16] The antibacterial activity of cell-free supernatants (CFS), obtained after cultivation of the LAB, was evaluated by the agar well diffusion method.[17] The CFS were obtained after centrifugation (10,000 g, 15 min) of 24 h LAB cultures in MRS and M17 broth (for lactobacilli and lactococci, respectively) cultivated at 28 °C for 24 h. CFSs were filter-sterilized and kept at 4 °C. In order to eliminate the action of lactic acid on the test-bacteria the pH of portions of the supernatants was adjusted to 6.0 with 6 mol/L NaOH. The sensitivity of the active substances to proteolytic enzymes and heat was estimated by treatment of the neutralized supernatants with proteinase K (0.2 mg/mL, Boehringer, Mannheim, Germany) and heating at 60 and 80 °C for 10 min, respectively. Nine different Gram-positive and Gram-negative test-bacteria – *Salmonella enteritica* serotype Enteritidis, *Salmonella enteritica* serotype Choleraesuis NBIMCC 2333, *Propionibacterium acnes* KPA, *Propionibacterium acnes* PA 266, *Listeria innocua* F (ONIRIS, Nantes, France*) Bacillus subtilis* NBIMCC 2353 (ATCC 6051), *Escherichia coli* NBIMCC 3397, *E. coli* NBIMCC 3548 and *Stapyloccocus epidermidis* NBIMCC 1093 were used in this study. The antibacterial activity of all variants CFSs (native, neutralized, treated with proteinase K and heated) was detected as inhibitory zones around the wells in the agar, inoculated with the test-bacteria.

## Results and discussion

### Enumeration and isolation of LAB

Six cultivated snails *C. aspersum* were investigated in this study. The snails in different stages in the life cycle were collected from private farms located in different regions of Bulgaria. The total number of LAB enumerated on MRS and M17 medium was higher (10^8^ CFU/mL) in breeding conditions than during hibernation (10^5^ CFU/mL).

Of about 350 colonies that were formed on the two medium, 122 small, round and opaque, and white colonies showed characteristics of LAB: Gram-positive non-spore-forming rods or cocci, catalase and oxydase negative, and aerotolerant. Fifty-five presumptive LAB strains were purely isolated and were phenotypically characterized (Table 1). Thirty-nine of the strains were homofermentative and 15 isolates – heterofermentative.

**Table 1.** Phenotypical characterization of the most representative lactic acid bacteria isolated from snail's gut.

Isolates	Characters	Tentative identification
Р1/24, P1/1, P1/14, P1/26, P1/27, P128, P1/22	Glycerol (−), Erythritol (−), D-Arabinose (−), L-Arabinose (+), D-Ribose (+), D-Xylose (+), L-Xylose (−), D-Adonitol (−), β-Methyl-D-xylopyranoside (−), D-Galactose (+), D-Glucose (+), D-Fructose (+), D-Mannose (+), L-Sorbose (−), L-Rhamnose (−), Dulcitol (−), Inositol (−), D-Mannitol (+), D-Sorbitol (−), α-Methyl-D-mannopyranoside (−), α-Methyl-D-glucopyranoside (−), N Acetyl glucosamine (+), Amygdaline (+), Arbutine (+), Esculine (+), Salicine (+), D-Cellobiose (+), D-Maltose (+), D-Lactose (−), D-Melibiose (−), D-Saccharose (+),D-Trehalose (+), Inuline (−), D-Melizitose (−), D-raffinose(−),Amidon (−), Glycogene (−), Xylitol (−), β-Gentiobiose (+), D-Turanose (−), D-Lyxose (−), D-tagatose (−), D-Fucose (−), L-Fucose(−), D-Arabitol (−), L-Arabitol (−), Potassium Gluconate (+), Potassium 2 keto-gluconate (−), Potassium 5 keto-gluconate (−)	*L. brevis* 90%
Р1/30, Р4/4	Glycerol (−), Erythritol (−), D-Arabinose (−), L-Arabinose (−), D-Ribose (+), D-Xylose (−), L-Xylose (−), D-Adonitol (−), β-Methyl-D-xylopyranoside (−), D-Galactose (+), D-Glucose (+), D-Fructose (+), D-Mannose (+), L-Sorbose (−), L-Rhamnose (−), Dulcitol (−), Inositol (−), D-Mannitol (+),D-Sorbitol (+), α-Methyl-D-mannopyranoside (−), α-Methyl-D-glucopyranoside (+), N Acetyl glucosamine (+), Amygdaline (+), Arbutine (+), Esculine (+), Salicine (+), D-Cellobiose (+), D-Maltose (+), D-Lactose (+), D-Melibiose (−), D-Saccharose (+), D-Trehalose (+), Inuline (−), D-Melizitose (−), D-raffinose (−), Amidon (−), Glycogene (−), Xylitol (−), β-Gentiobiose (+), D-Turanose (+), D-Lyxose (−), D-tagatose (−), D-Fucose (−), L-Fucose (−), D-Arabitol (−), L-Arabitol (−), Potassium Gluconate (+), Potassium 2 keto-gluconate (−), Potassium 5 keto-gluconate (−)	*L. plantarum* 99.9%
P1/15, Р2/30, Р3/1 Р3/5, Р3/6, Р4/1, Р4/2, Р4/5, Р4/6, Р4/10, Р4/12, Р5/15 Р7/4	Glycerol (−), Erythritol (−), D-Arabinose (−), L-Arabinose (+), D-Ribose (+), D-Xylose (+), L-Xylose (−), D-Adonitol (−), β-Methyl-D-xylopyranoside (−), D-Galactose (+), D-Glucose (+), D-Fructose (+), D-Mannose (+), L-Sorbose (−), L-Rhamnose (−), Dulcitol (−), Inositol (−), D-Mannitol (−), D-Sorbitol (−) α-Methyl-D-mannopyranoside (−), α-Methyl-D-glucopyranoside (−), N Acetyl glucosamine (+), Amygdaline (+), Arbutine (+), Esculine (+), Salicine (+), D-Cellobiose (+), D-Maltose (+), D-Lactose (+), D-Melibiose (−), D-Saccharose (+), D-Trehalose (+), Inuline (−), D-Melizitose (−), D-raffinose(−), Amidon (−), Glycogene (−), Xylitol (−), β-Gentiobiose (+), D-Turanose (−), D-Lyxose (−), D-tagatose (−), D-Fucose (−), L-Fucose(−), D-Arabitol (−), L-Arabitol (−), Potassium Gluconate (+), Potassium 2 keto-gluconate (−), Potassium 5 keto-gluconate (−)	*L. lactis* ssp. *lactis* 97.0%
P4/11	Glycerol (−), Erythritol (−), D-Arabinose (−), L-Arabinose (+), D-Ribose (+), D-Xylose (+), L-Xylose (−), D-Adonitol (−), β-Methyl-D-xylopyranoside (−), D-Galactose (+), D-Glucose (+), D-Fructose (+), D-Mannose (+), L-Sorbose (−), L-Rhamnose (−), Dulcitol (−), Inositol (−), D-Mannitol (−), D-Sorbitol (−), α-Methyl-D-mannopyranoside (−), α-Methyl-D-glucopyranoside (−), N Acetyl glucosamine (+), Amygdaline (+), Arbutine (+), Esculine (+), Salicine (+), D-Cellobiose (+), D-Maltose (+), D-Lactose (−), D-Melibiose (−), D-Saccharose (+), D-Trehalose (+), Inuline (−), D-Melizitose (−), D-raffinose(−), Amidon (−), Glycogene (−), Xylitol (−), β-Gentiobiose (+), D-Turanose (−), D-Lyxose (−), D-tagatose (−), D-Fucose (−), L-Fucose (−),D-Arabitol (−), L-Arabitol (−), Potassium Gluconate (+), Potassium 2 keto-gluconate (−), Potassium 5 keto-gluconate (−)	*Weissella confusa* 63.7%
P5/4, P5/8, P5/9	D-Glucose (+), D-mannitol (−), Inositol (−), D-sorbitol (−), L-rhamnose (−), D-sucrose (−), D-melibiose (−), Amygdalin (−), L-arabinose (+/-), Ribose (acid) (−), Glucose (gas) (−),Galactose (−), Raffinose (−), Cellobiose (−), Manosse (+), Maltose (−), Xylose (−), Fructose (+), Dextrose (+)	*L. curvatus 91% L.sakei 87%*
P5/5	D-Glucose (+), D-mannitol (−), Inositol (−), D-sorbitol (−), L-rhamnose (−), D-sucrose (−), D-melibiose (−), Amygdalin (−), L-arabinose (+/-), Ribose (acid) (−), Glucose (gas) (−), Galactose (−), Raffinose (−), Cellobiose (−), Manosse (+), Maltose (+), Xylose (−), Fructose (+), Dextrose (+)	*L. graminis 89%*
P2-18	Pyruvat (+), Hippurat (−), Esculin (+), Pyrrolidonyl-2-naphthylamide (+), 6-Bromo-2-naphtyl α-D-galctopyranoside (−), Naphthol AS-BI β-D-glucuronate (−), 2-naphthyl-β-D-galactopyranoside (+), 2-naphtyl phosphate (−), L-leucine-2-naphtylamide (+), Arginine (+/−), Ribose (+), L-arabinose (+), Mannitol (+), Sorbitol (−), Lactose (+/−), Trehalose (+), Inulin (+/−), Raffinose (+/−), Starch (+/−), Glycogen (−)	*E. faecium E. casseliflavus*
P/2-19	Pyruvat (+), Hippurat (−), Esculin (+), Pyrrolidonyl-2-naphthylamide (−), 6-Bromo-2-naphtyl α-D-galctopyranoside (−), Naphthol AS-BI β-D-glucuronate (−), 2-naphthyl-β-D-galactopyranoside (+), 2-naphtyl phosphate (−), L-leucine-2-naphtylamide (+), Arginine (+), Ribose (+), L-arabinose (+), Mannitol (+), Sorbitol (+), Lactose (+), Trehalose (+), Inulin (−), Raffinose (+), Starch (+/-), Glycogen (−)	*E. faecium E.mundtii*

Notes: +: positive; w+: weak positive; −: negative.

### Molecular differentiation of LAB

**Figure 1.**
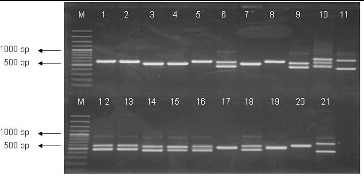
PCR-amplification of 16S-23S ITRs (16S-4/23S-7 primers) of LAB strains. Lane 1: strain P3/3, lane 2: P3/4, lane 3: P3/5, lane 4: *L. lactis* 454, lane 5: P3/8, lane 6: P3/9, lane 7: P4/1, lane 8: P4/8, lane 9: P4/9, lane 10: P4/11, lane 11: P1/30, lane 12: P2/4, lane13: *Enterococcus faecium*, lane 14: P2/18, lane 15: P2/19, lane 16: P2/24, lane 17: P4/6, lane 18: P2/32, lane 19: P4/2, lane 20: *Ln mesenteroides* ATCC 8293 and lane 21: *L. plantarum* NBIMCC 297, M-molecular weight marker (100-bp DNA ladder, Fermentas).

All purified strains were subjected to a preliminary molecular characterization using the 16S-23S ITS PCR approach and were differentiated at the genus level. The results of the amplifications are shown in Figure 1. Thirty-five strains formed amplification pattern with two fragments. The larger fragment was the same in all studied strains – about 650 bp, whereas the smaller fragment was about 450 bp in 18 and about 550 bp in 17 of these strains. Seven isolates showed the profile with one major band of approximately 600 bp, 12 isolates – one fragment of 500 bp and one LAB isolate – three fragments (450, 550, 650 bp). Comparing our results with the literature data,[10] it could be concluded that 18 of our isolates belonged to the genus *Lactobacillus*, 17 – to the genus *Enterococcus*, 7 – to the genus *Leuconostoc*, 12 – to the genus *Lactococcus*, and one strain – to the genus *Weissella*, as well they formed characteristic amplification pattern to the corresponding genera.

### Identification of lactobacilli

**Figure 2.**
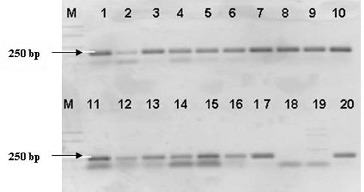
PCR-amplification of *Lactobacillus* strains with genus specific primer LBMA1. Lane 1: strain P1/1, lane 2: strain P1/2, lane 3: strain P1/3, lane 4: strain P1/4, lane 5: strain P1/5, lane 6: strain P1/14, lane 7: strain P1/22, lane 8: strain P1/24, lane 9: strain P1/26, lane 10: strain P1/27; lane 11: strain P1/28, lane 12: strain P5/4, lane 13: strain P5/5, lane 14: strain P5/8, lane 15: strain P5/9, lane 16: : *L. plantarum* NBIMCC 297, lane 17: *L.fermentum* NBIMCC 505, lane 18: *Ln mesenteroides* ATCC 8293, lane 19: *L. lactis* 454 and lane 20: *L. plantarum* 117. M-molecular weight marker (100-bp DNA ladder, Fermentas).

The confirmation of the affiliation of the strains identified by 16S-23S ITS PCR as member of the genus *Lactobacillus* was performed with lactobacilli genus-specific primer (LbLMA1). All 18 strains amplified an expected fragment of about 250 bp [18]. These results proved their belonging to the genus *Lactobacillus* (Figure 2).

Since lactobacilli are extremely heterogeneous, they were separated into groups according Song et al.[11] Therefore, we applied a two-step multiplex PCR to differentiate our isolates into previously described groups. The first multiplex PCR was performed with the four pairs of group specific primers, namely Ldel-7/Lac 2, Lu1/Lac2, Lu-5/Lac2 and Lu-3/Lac2. This approach separated the lactobacilli into four groups: Group I (*Lactobacillus delbrueckii* ssp. *bulgaricus*/*lactis)*, Group II (*Lactobacillus acidophilus*, *Lactobacillus helveticus*, *Lactobacillus amylovorus*, *Lactobacillus crispatus*, *Lactobacillus gasseri*, *Lactobacillus johnsonii)*, Group III (*Lactobacillus paracasei*, *Lactobacillus casei* and *Lactobacillus rhamnosus*.) and Group IV (*L. plantarum* and *Lactobacillus fermentum),* respectively.[11] The obtained results showed that only the primers pair for Group IV (Lu-3/Lac2) gave the positive reaction for six of our strains (P1/30, P4/4, P5/4, P5/5, P5/8 and P5/9) forming an expected amplification band – 350 bp. These strains were subjected on a second multiplex PCR with species-specific primers for this group, and two of them (P1/30 and P4/4) were identified as *L. plantarum*. The four strains (P5/4, P5/5, P5/8 and P5/9) which were not identified by a two-step multiplex PCR showed similarity by fermentation patterns (API CH50) to the species *Lactobacillus curvatus* (91%), *Lactobacillus graminis* (89%) or *Lactobacillus sakei* (87%) (Table 1).

The rest of the investigated strains formed an amplicon of 500 bp which did not correspond to any of the described group. API 50CH fermentation profiles of these strains revealed the similarity to *Lactobacillus brevis* (90%) that was not confirmed by species-specific PCR.

**Figure 3.**
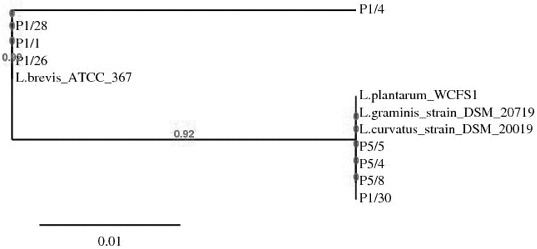
Phylogenetic tree constructed by the platform http://www.phylogeny.fr (8) based on sequences of 16S rDNA of selected LAB strains associated snail-gut: *L.plantarum* P1/30, *L.curvatus* P5/4, *L.curvatus* P5/8. *L.graminis* P5/5, *L.brevis* P1/1, *L.brevis* P1/26, *L.brevis* P1/28 and GenBank available sequences: *L.brevis* ATCC 367, *L.curvatus* DSM 20019 and *L.graminis* DSM 20719.

Selected *Lactobacillus* strains analysed by the above-applied methods were subjected to 16S rDNA sequence analysis. Comparison of the obtained sequences with the corresponding *Lactobacillus* sequences available in GenBank database showed that all 14 investigated strains laid in the evolutionary clade of the *Lactobacillus*. Eight strains had 99%–100% identity with *L. brevis* ATCC 367, three strains showed 99% homology score to *L. curvatus* DSM 20019, one strain was identified with 99% similarity to *L. graminis* DSM 20719 and two strains were 100% identical with *L. plantarum* WCFS1. A phylogenetic tree of lactobacilli (determined in this study) was constructed using the neighbour-joining algorithms,[19,20] by comparing the available sequences (NCBI). The phylogenetic relationships based on 16S resulted in the separation of isolated lactobacilli strains into two group – group I *brevis* and group II *plantarum – curvatus – graminis* (Figure 3).

### Identification of cocci

**Figure 4.**
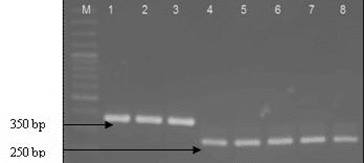
Multiplex PCR identification of strains belonging to species *Lactococcus lactis* and *Leuconostoc mesenteroides*. Lane 1: *Ln mesenteroides* ATCC 8293, lane 2: Р3/8, lane 3- Р4/8, lane 4 - *Lactococcus lactis* ssp. *lactis* 454, lane 5- P 3/1, lane 6 - P 3/5, lane 7- P3/6 and lane 8- P4/1, M- molecular weight marker (100-bp DNA ladder, Fermentas).

The isolates identified to the genus *Lactoccoccus* (12) and *Leuconostoc* (7) on the basis of 16S-23S ITS rDNA were subjected to species-specific multiplex PCR.[12] All *Lactococcus* isolates gave a positive reaction with primers specific for *L. lactis*, forming an amplification product of about 248 bp. Four *Leuconostoc* isolates were identified as *Leuconostoc mesenteroides* on the base of resulted amplification fragment of about 358 bp (Figure 4). For the all 17 *Enterococcus* strains the PCR amplification with species-specific primers (CA1/CA2, Efm1/Efm2 and Efs1/Efs2) was applied. Only one isolate (P4/9) was identified as *E. casseliflavus*. It should be noted that the test of the *Enterococcus* strains based on the API Strep 20 showed similarity to the species of *faecium* group. Our result confirmed the finding of Charier et al. [1] that *E. casseliflavus* actually reside in snail gut. The authors also found that the lactococci occurred in *H. pomatia* gut, while the enterococci were present in French populations of both species (*H. pomatia* and *C. aspersum)*. Our results extend the available information on the spread of lactococci in the snail species.

### Antibacterial assessment

Antimicrobial activity of whole cells of isolated *Lactobacillus* (13 strains), *Lactococcus* (12 strains) and *Leuconostoc* (3 strains) strains were screened against four test-bacteria: *S. enteritica* serotype Enteritidis, *S. enteritica* serotype Choleraesuis, *B. subtilis* and *S. epidermidis*. The highest activity of lactobacilli (inhibition zone – 25–30 mm) was observed against *S. enteritica* serotype Enteritis, *S. enteritica* serotype Choleraesuis and *S. epidermidis*. The activity of *Lactococcus* and *Leuconostoc* strains varied.

**Figure 5.**
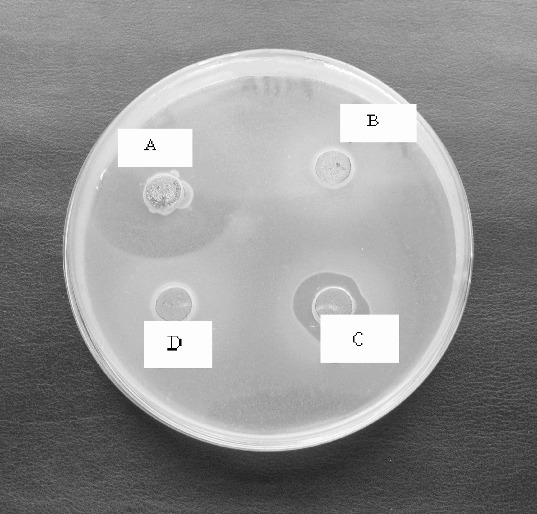
Antibacterial activity of neutralized cell free supernatant (NCFS) from *Leuconostoc mesenteroides* P4/8 against *Propionibacterium acnes*: A- NCSF pH 6.0, B – NCFS treated with Proteinase K, C- NCFS treated at 60 °C for 10 min, D- NCFS treated at 80 °C for 10 min.

The native CFS of the strains showed antibacterial activity against *S*. serotype Enteritis, *S*. serotype Cholaeresuis, *P. acnes, L. innocua F*., *B. subtilis*, *E.coli*, and *S. epidermidis* forming a relatively small inhibition zones (10–11 mm). Only two *Leuconostoc* isolates (*L. mesenteroides* P4/8 and P3/2) showed antibacterial activity of the neutralized CFS (pH 6.0) especially against *P. acnes*. This activity was reduced after heating at 80 °C for 10 min and disappeared after proteinase K treatment (Figure 5). It could be suggested that the ability of the *L. mesenteroides* P4/8 and P3/2 to inhibit *P. acnes* strains may be due to the production of heat labile bacteriocin-like substance.

## Conclusions

The present study confirmed that LAB are endogenous and could be assigning to ‘enteric’ environment. For the first time by molecular approach, the presence of the species *L. brevis*, *L. plantarum*, *L. curvatus*, *L.graminis* and *L. mesenteroides* was determined. This investigation is the first molecular typing of LAB associated with the gut of the snail *C. aspersum*.
